# Public health round-up

**DOI:** 10.2471/BLT.15.010315

**Published:** 2015-03-01

**WHO tobacco treaty marks its 10th anniversary**

The image of cigarette packs has changed dramatically since the WHO Framework Convention on Tobacco Control (FCTC) entered into force in February 2005. This photograph shows one of the new mandatory health warnings on a packet of cigarettes in the Russian Federation, one of many countries that have passed tough tobacco control legislation in line with the FCTC.

**Figure:**
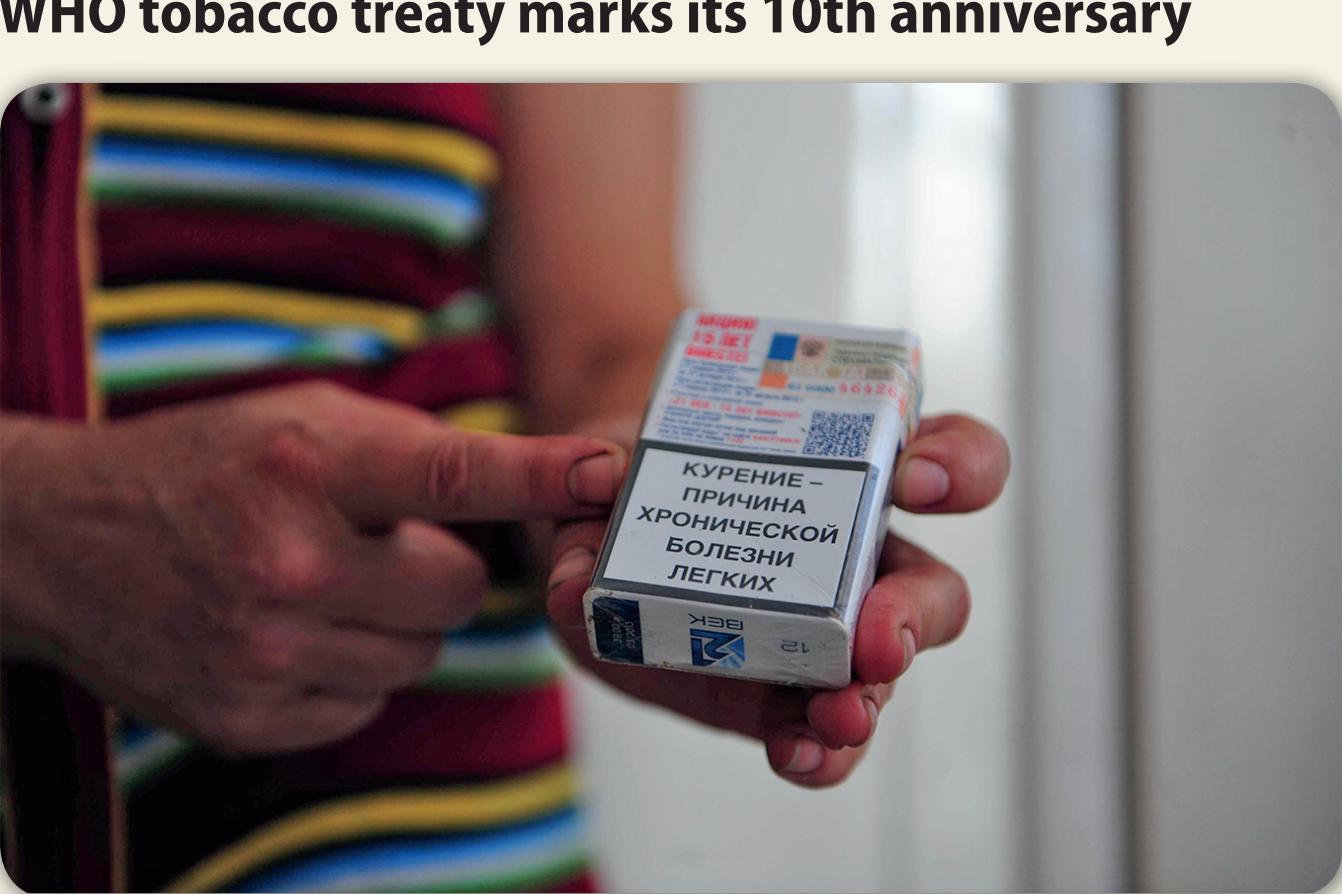
WHO/V Ivleva

## Ebola special session

At a Special Session of the Executive Board on the Ebola outbreak in West Africa, Member States agreed to establish a contingency fund for the World Health Organization’s (WHO) work in emergencies and outbreaks and to draw up plans for a global health emergency workforce.

The Executive Board also recommended the appointment of a Special Representative of the Director-General for the Ebola Response, who will be responsible for all aspects of coordination at the three levels of the Organization and for the response to the current outbreak.

Director-General Dr Margaret Chan subsequently appointed Dr Bruce Aylward to the position with immediate effect, and for the duration of the outbreak.

The Special Session on 25 January adopted resolution EBSS3/EBSS3_R1, which provides guidance on the immediate steps WHO and Member States should take to end the outbreak and sets out an agenda for the reform of WHO’s work in outbreaks and emergencies.

The proposed emergency workforce is vital “to ensure that the world has a reserve force of teams of health workers and other responders, trained and experienced in health emergencies, and able to be rapidly mobilized in the event of a health emergency,” Chan told WHO staff in a message.

“This emergency workforce will comprise teams of WHO staff from all three levels of the Organization, an expanded Global Outbreak Alert and Response Network, foreign medical teams, United Nations staff, and other groups,” she said.

Currently almost 700 WHO staff and consultants are deployed in Guinea, Liberia and Sierra Leone, the three countries most affected by the outbreak.

The Executive Board also asked the Director-General to commission an interim evaluation of WHO’s response to the outbreak by a panel of independent experts.

The results of the evaluation and the plans for the global health emergency workforce will be submitted to the World Health Assembly in May.

“This evaluation, and the subsequent review proposed to be carried out by the International Health Regulations Review Committee, will help to guide our future work in emergencies and outbreaks, including reforms of the structures and management systems that will enable us to most effectively fulfil our mandate in this critically important aspect of the work of WHO,” the Director-General said.

http://apps.who.int/gb/ebwha/pdf_files/EBSS3/EBSS3_R1-en.pdf

## US$ 7.5 billion for vaccines

Donor countries and private foundations pledged a record US$ 7.5 billion to Gavi, the Vaccine Alliance, over the next five years (2016–2020) at a conference hosted by the current G7 chair, Germany, in Berlin on 27 January.

The US$ 7.5 billion will be combined with US$ 2 billion in previously pledged funds to help to immunize an additional 300 million children in the world’s poorest countries from 2016 to 2020.

Since the organization was formed in 2000, more than half a billion children have been immunized with support from Gavi and, thus, more than seven million deaths have been averted.

The new funds provide a much-needed boost for the Decade of Vaccines’ Global Vaccine Action Plan (GVAP) that aims to prevent millions of deaths by 2020 through more equitable access to vaccines.

“Gavi, the Vaccine Alliance, is an innovative public–private partnership that has delivered on its promise and continues to demonstrate results, value for money and return on investment,” said Dr Flavia Bustreo, WHO Assistant Director-General for Family, Women’s and Children’s Health and Vice Chair of the Gavi Board.

“It has done so by building from WHO’s unique role in providing evidence-based guidance including setting immunization policies and international standards to guide vaccine use in countries and supporting health system development to enable the integration of other preventive interventions as part of comprehensive approaches to public health,” she said.

A 2014 assessment report of the GVAP by WHO’s principal advisory group on vaccines and immunization, the Strategic Advisory Group of Experts on Immunization, concluded that progress towards global immunization targets was slow, citing weak implementation as a major barrier.

The GVAP was endorsed by the World Health Assembly in May 2012 to achieve its Decade of Vaccines’ vision of universal access to immunization.

http://www.gavi.org/Funding/Resource-mobilisation/Process/Gavi-pledging-conference-January-2015

**Cover photo**

A health worker records the weight of an infant to monitor its growth at the Ourika Health Centre. The centre, located near Marrakesh in Morocco, is supported by the United Nations Children’s Fund (UNICEF).

**Figure:**
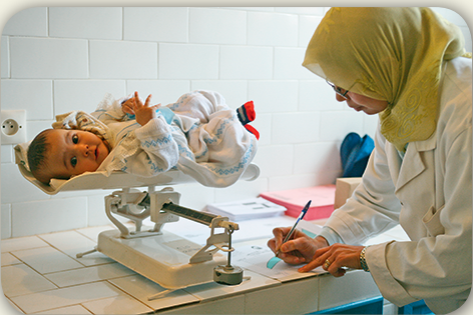
UNICEF/G Pirozzi

## WHO office in east Ukraine

A WHO field office opened in the city of Donetsk in crisis-hit eastern Ukraine last month to respond better to the health needs of the people and coordinate the many humanitarian organizations providing emergency health services there.

More than a million people have been displaced and hundreds killed in the conflict, and many health facilities have been damaged, abandoned or relocated.

The medical referral system has been disrupted, and – as casualties mount and health facilities close – hospitals are under increased pressure to take in more patients.

The interruption of financial support to these hospitals has jeopardized payment of medical staff salaries and the supply chain of medicines has collapsed.

“We urgently need to get medicines for chronic diseases – such as tuberculosis, HIV infection, cancer and diabetes – to the region,” said Dr Dorit Nitzan, the WHO Representative to Ukraine.

“Our international office field coordinator is working with health-cluster partners to assess availability of and access to health services and monitor the health status and needs of different population groups,” Nitzan said.

The new field office is part of the joint United Nations humanitarian team’s network of field offices in the country. WHO teams in these offices are supervised by the WHO Country Office.

http://www.euro.who.int/en/countries/ukraine/news/news/2015/02/who-strengthens-health-operations-in-eastern-ukraine

## Neglected no more

Countries should invest more in the prevention and control of neglected tropical diseases, especially low-income countries that will soon make the transition to middle-income status, according to a new WHO report released last month.

The report, *Investing to overcome the global impact of neglected tropical diseases,* outlines what is needed to tackle 17 neglected tropical diseases affecting more than a billion people in 149 countries worldwide and sets out financing needs for meeting the WHO Roadmap goals by 2020 on these diseases.

http://www.who.int/gho/neglected_diseases

## WHO conference on dementia

Health officials from around the world will join experts from the research, clinical and nongovernmental communities at WHO headquarters in Geneva from 16 to 17 March to discuss the global problems posed by dementia.

Many countries are already facing the challenge of providing health and social care for their ageing populations.

The aim of the first WHO Ministerial Conference on Global Action Against Dementia is to raise awareness of the socioeconomic burden created by dementia – a significant problem associated with ageing – and to highlight the need to reduce this burden and to place dementia high on the global political agenda.

http://www.who.int/topics/dementia

## Attacks on health workers

The World Health Organization called on all parties in countries experiencing conflict to “respect and protect the integrity of health systems, and assure the safety of patients, health workers and health care facilities,” in a statement issued by the WHO Office for the Eastern Mediterranean Region on 29 January.

“The World Health Organization deplores the recent attacks on health staff and health facilities in Afghanistan, Sudan and Yemen, and expresses deep concern about the serious implications of these attacks on patients, medical personnel and health infrastructure,” the statement said.

http://www.emro.who.int/media/news/who-deplores-attacks-on-health-facilities.html

## Second term for Jakab

Dr Zsuzsanna Jakab, started her second five-year term in office as WHO Regional Director for Europe after her appointment by WHO’s Executive Board in January. She was nominated for the post by the WHO Regional Committee for Europe in September.

A native of Hungary, Jakab has held several high-profile national and international public health policy positions over the last three decades, including as the founding Director of the European Union’s European Centre for Disease Prevention and Control in Stockholm, Sweden.

http://www.euro.who.int/en/about-us/regional-director/biography

**Looking ahead**

**20 March – World Oral Health Day**. http://www.worldoralhealthday.com/fdi-launches-its-world-oral-health-day-2015-smile-for-life-campaign
**22 March – World Water Day.** This year marks the end of the International Decade for Action: Water for Life 2005–2015. http://www.who.int/water_sanitation_health/decade2005_2015
**7 April – World Health Day.** This year’s theme is food safety. http://www.who.int/campaigns/world-health-day/2015/event

